# Intelligent Malaysian Sign Language Translation System Using Convolutional-Based Attention Module with Residual Network

**DOI:** 10.1155/2021/9023010

**Published:** 2021-12-10

**Authors:** Rehman Ullah Khan, Hizbullah Khattak, Woei Sheng Wong, Hussain AlSalman, Mogeeb A. A. Mosleh, Sk. Md. Mizanur Rahman

**Affiliations:** ^1^Faculty of Cognitive Sciences and Human Development, Universiti Malaysia Sarawak, Kuching, Sarawak 94300, Malaysia; ^2^Department of Computer Science & Information Technology, Hazara University Mansehra, Mansehra, 21120 Khyber Pakhtunkhwa, Pakistan; ^3^Department of Computer Science, College of Computer and Information Sciences, King Saud University, Riyadh 11543, Saudi Arabia; ^4^Faculty of Engineering and Information Technology, Taiz University, Taiz 6803, Yemen; ^5^Information and Communication Engineering Technology, School of Engineering Technology and Applied Science, Centennial College, Toronto, Canada

## Abstract

The deaf-mutes population always feels helpless when they are not understood by others and vice versa. This is a big humanitarian problem and needs localised solution. To solve this problem, this study implements a convolutional neural network (CNN), convolutional-based attention module (CBAM) to recognise Malaysian Sign Language (MSL) from images. Two different experiments were conducted for MSL signs, using CBAM-2DResNet (2-Dimensional Residual Network) implementing “Within Blocks” and “Before Classifier” methods. Various metrics such as the accuracy, loss, precision, recall, *F*1-score, confusion matrix, and training time are recorded to evaluate the models' efficiency. The experimental results showed that CBAM-ResNet models achieved a good performance in MSL signs recognition tasks, with accuracy rates of over 90% through a little of variations. The CBAM-ResNet “Before Classifier” models are more efficient than “Within Blocks” CBAM-ResNet models. Thus, the best trained model of CBAM-2DResNet is chosen to develop a real-time sign recognition system for translating from sign language to text and from text to sign language in an easy way of communication between deaf-mutes and other people. All experiment results indicated that the “Before Classifier” of CBAMResNet models is more efficient in recognising MSL and it is worth for future research.

## 1. Introduction

Malaysia Sign Language (MSL), or Bahasa Isyarat Malaysia in Malay, was founded in 1998 when the Malaysia Federation of the Deaf (MFD) was established [1]. It is the primary sign language in Malaysia. It is used for daily communication for the deaf-mute community, including deaf people, people with hearing impairments, and physically unable to speak. The MSL has grown in popularity among deaf leaders and participants. Generally, the American Sign Language (ASL) has had a significant influence on Malaysian Sign Language. Although there are a few similarities between the MSL and Indonesian Sign language, both are perceived differently. Otherwise, the foundation of Indonesian Sign language was based on MSL. The communication is accomplished by interpreting the meaning of the signer's hand gestures and, on occasion, by using appropriate facial expressions. In 2013, about 58700 people from the Malaysian population used the MSL [2].

Most of the time, the intended meaning that deaf-mutes wish to deliver throughout interaction were often misunderstood or hard to comprehend by others. Many ordinary people are not familiar with and cannot understand MSL. To master sign language is very challenging and highly depends on a person's willingness to learn. If people do not understand the MSL, they will confront problems communicating with the deaf-mutes in the nation. The implications of ineffective communication had affected the deaf-mutes in their psychological, educational, employment, and social dimensions. Every one of us will need a good listener to share our feelings and thoughts. The students with hard of hearing resulted in more mental health issues than their peers who can hear [3]. Deaf people at different age groups were often related to higher distress, somatisation, and feeling lonely and depressed [4]. These feelings may be due to their failure in interpersonal communication. In higher education, the chances of deaf-mutes interacting with teachers or lecturers may be less than others because of incommunicability. It can suppress and affect their learning experience in class. It was difficult for deaf-mutes to seek employment in Malaysia due to their inabilities in hearing and speech. A study carried out by Khoo et al. revealed a few cases of job discrimination, bullying, and exploitation of individuals with hearing impairment in Malaysia [5]. Sometimes, negative emotions experienced by deaf-mutes made them feel ostracised by society because they were not being understood.

Before emerging sign language translation software or applications, human interpreters have relied on it as the communication bridge between deaf-mutes and people in different fields. The availability of a professional human interpreter to aid in translating sign language has become an issue as it involves considering cost and time to users. There is no efficient machine learning–based sign language translation software for MSL to convert the sign into sentences or voice for public usage. These issues had caused the communication problems or gap among the deaf-mute individuals and the society. The talented deaf-mute individuals cannot present their ideas to others. As a result, the society is not only losing the valuable talent, but the deaf-mute individuals are getting mental problems due to this gap. Although much research on MSL recognition that has been conducted in the past had achieved good accuracy of recognition, most of them were only focused on static-type sign language that tested with limited vocabulary. It was insufficient for a daily-use language to help the deaf-mutes in their communication with others. The findings from these studies lacked robustness in developing the efficient MSL translating mechanism. There is an open research area for the development of MSL recognition related technologies. Therefore, there is need of real-time sign language translation system to fill the communication gap among the deaf-mutes and the community.

Hence, this research on sign language recognition contributes to the technology used to remove communication barriers between these populations and other people. The introduction of the CBAM-ResNet method in this paper fills up the research gap in MSL recognition technology. CBAM [6] consists of a channel and spatial attention submodules, which are used to extend the structure and enhance the performance of Residual Network (ResNet) in images recognition. This study emphasised this method's performance, efficiency, and practicability to produce a robust sign language translation system that benefits Malaysian deaf-mutes. CBAM-ResNet 2D convolutions are implemented with two methods known as “Within Blocks” and “Before Classifier.” Efficiency evaluation of CBAM-ResNet was completed using multiple metrics such as classification accuracy, loss, precision, recall, *F*1-score, confusion matrix, and training time.

### 1.1. Significance and Contribution

The main objective of this research is to test and evaluate the efficiency of the CBAM-ResNet method using MSL. The School of Automation initially conducted CBAM-ResNet neural network and Electrical Engineering (USTB) of Beijing, China, to implement Chinese Sign Language Recognition, which has a different network architecture from this study [7]. As sign languages vary from place to place, it is crucial to determine the diversification and performance of model in terms of multi-metric before it can be widely implemented on MSL. Therefore, this study designed a new model and combined CBAM with ResNet to extend the structure and enhance the performance of ResNet. The subobjectives of this research are as outlined as follows:

- To implement the new method, which is CBAM-ResNet on MSL recognition, to increase the efficiency of the sign language recognising mechanism.
- To further investigate the differences between CBAM-ResNet “Within Blocks” and “Before Classifier” regarding the efficiency of recognising MSL.
- To develop a real-time MSL Recognition System through human gestures recognition using the CBAM-ResNet method.

This is the first study that adopts the CBAM-ResNet method in the context of MSL. This study introduces the CBAM-ResNet neural network to resolve the problems such as accuracy and applicability in the previous MSL recognition technology. By evaluating the efficiency of the CBAM-Resnet method on MSL recognition, the proposed method can be effective as the researcher expectation and helps identify any potential for improving the sign language translating mechanism. This study is also crucial to improve communication between deaf-mute's populations and ordinary people in Malaysia to understand each other throughout their conversations. Once the efficiency of CBAM-ResNet on MSL recognition is proven, it can be implemented to develop a robust MSL translating system. Thus, deaf-mutes will enjoy equal access to the same privileges as ordinary people and encourage social harmony.

### 1.2. Organisation

The remainder of the work is organised as follows: Section 2 briefly discusses related past studies on other sign languages recognition. The methodology of CBAM-ResNet implementation on MSL is explained in Section 3.  Section 4 presents the experimental settings, results, and discussion to compare CBAM-ResNet “Within Blocks” and “Before Classifier” in MSL signs recognition. Finally, Section 5 provides the conclusion for this paper.

## 2. Literature Review

From past till nowadays, various computational algorithms and machine learning methods have been applied to different sign language recognition, such as Artificial Neural Network (ANN), Convolution Neural Network (CNN), Support Vector Machine-based machine learning (SVM), Hidden Markov Model-based machine learning (HMM), Fuzzy rules-based algorithm, Back-Propagation algorithm, Recurrent Neural Network (RNN), 3D Residual Convolutional Network (3D-ResNet), and CBAMResNet neural network. These methods have their respective strengths and limitations in recognising sign languages. Generally, researchers in past studies used two main streams of sign languages recognition methods: vision-based and glove-based techniques. The vision-based method was relatively more convenient than the glove-based method as it does not require any wearable device, making it a hassle-free solution. However, the vision-based method still has limitations, such as the quality of the camera and image used, capturing distance and direction from the camera, lighting of surroundings, accessories worn by signers, and overlapping hands in presenting sign language [8–10]. These factors may affect the performance of the model. The critical evaluation parameters such as accuracy, speed of recognition, time of response, applicability, and accessibility are used to measure the efficiency of the sign language recognition algorithm.

### 2.1. Relevant past Studies on Different Sign Languages around the World

As the world is more concerned with deaf-mute's welfare, it shows positive development and a gradual increase in research associated with sign languages in recent times. Researchers worldwide have proposed different machine learning algorithms in sign languages recognition. Meanwhile, methods implemented on sign languages recognition also change with advancements in technology, which can boost the performance of those machine learning algorithms.

#### 2.1.1. Artificial Neural Network

An artificial neural network (ANN) consists of nodes, simulating the neurons interconnections in biological life's brain [11]. It was usually applied to solve the problems that required data processing and knowledge representation. For example, Tangsuksant et al. [12] researched American Sign Language static alphabets recognition using feedforward backpropagation of ANN. Their research returned an average accuracy of 95% throughout repeating experiments. Another study used the same method and achieved a higher average accuracy of 96.19%, with 42 letters of Thai Sign Language examined [13].

López-Noriega, et al. selected gloves with built-in sensors for sign alphabets recognition using ANN with Backpropagation, Quick propagation, and Manhattan propagation [14]. Mehdi and Khan [15] carried out a study with seven sensors equipped with gloves and ANN architecture, which had achieved an accuracy rate of 88%. Finally, Allen et al. [16] developed a fingerspelling recognition system using MATLAB for American Sign Language alphabets. The chosen neural network was perceptron which received a matrix with 18 rows and 24 columns as input from the 18 sensors on CyberGlove through training. Their model got an accuracy of 90%.

#### 2.1.2. Convolutional Neural Network (CNN)

Convolutional Neural Network is a subtype of the Feed-Forward Neural Network (FNN) suitable for images and videos processing [17]. Jalal et al. [18] proposed an American Sign Language translator that did not rely on pre-trained models. They proved that their model has up to 99% recognition accuracy. It was higher than the modified CNN model from Krizhevsky [19]. Another study that employed CNN on an American Sign Language dataset with around 35000 images was carried out in India [20]. This study adapted a CNN with the topology of three convolutional layers with 32, 64, and 128 filters, max-pooling layers, and Rectified Linear Unit (ReLu) activation function [21]. Through experimental testing, their proposed system was able to achieve 96% recognition accuracy.

#### 2.1.3. Recurrent Neural Network (RNN) and Long Short-Term Memory (LSTM)

A Recurrent Neural Network (RNN) is one of the neural networks equipped with internal memory, where its output will be mapped again into RNN for duplication. As RNN depends on inputs from previous sessions in the sequence, the duplicated antecedent elements will merge with the new input for completing decision-making tasks [22]. However, RNN usually has the problem of vanishing gradient in training. Therefore, Long Short-Term Memory (LSTM) is introduced as a refined version of RNN that can deal with this problem.

Liu et al. [23] suggested an LSTM-based Chinese sign language system with their self-build sign language vocabulary datasets using Microsoft Kinect 2.0. Their study returned an accuracy rate of 63.3%. Besides, RNN and LSTM are also applied in the sign language of Bahasa Indonesia with the use of TensorFlow [24]. They extend to recognising root words attached with affixes, which vary from the original meaning and parts of speech such as “noun” or “verb.” A study from Indonesia implemented the 1-layer, 2-layers, and bidirectional LSTM. It achieved 78.38% and 96.15% of recognition accuracy on inflectional words and root words.

All efforts contributed by researchers in previous studies on exploring robust sign language recognition mechanisms are much appreciated.

### 2.2. Relevant past Studies on MSL

**Figure 1:**
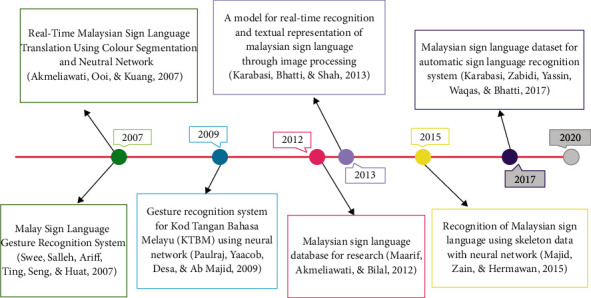
The timeline diagram for past studies related to Malaysian sign language.

The timeline diagram of some published studies on the MSL through the past 13 years is depicted in Figure 1. It shows the trends of researches in the field of this study. For example, Akmeliawati et al. [25] proposed an automatic sign language translator to recognise only fingerspelling and sign gestures. Another gesture recognition system for Malay sign language collected inputs from 24 sensors, consisting of accelerometers and flexure sensors connected via Bluetooth module wirelessly [26]. These studies could not provide real-time translation system.

A gesture recognition system was developed for Kod Tangan Bahasa Melayu (KTBM) in 2009. It captured images through a webcam and then processed with Discrete Cosine Transform (DCT) to produce feature vectors [27]. This system obtained 81.07% for classification rate using an ANN model. In 2012, researchers for MSL built a well-organised database with different classifications [28]. In the consequent year, Karabasi et al. [29] proposed a model for a signs recognition system through a mobile device in real-time. Majid et al. [1] implemented ANN with Backpropagation to classify skeleton data of signs obtained from Kinect sensors. They trained the network with a learning rate of 0.05 using 225 samples and achieved 80.54% accuracy on 15 dynamic signs. In 2017, Karbasi et al. [30] demonstrated a dataset development for MSL consisting of alphabets and ten (10) dynamic signs using Microsoft Kinect. In 2019, Fahmi et al. [31] proposed a hand signs translator system based on the fuzzy logic method. The system translates the hand patterns into A-Z English alphabet. Also, the use of the fuzzy logic method has the advantage to deal with uncertain cases of the input and unknown parameters of the system [32, 33]. All these studies could not solve the problem of translation of gestures into text and voice.

Researchers favoured ANN in recognising Malaysia Language signs. Therefore, this study implemented a CNN-based neural network called CBAM-ResNet, introducing a new classification method in MSL recognition to solve the gestures translation problem.

## 3. Methodology

### 3.1. Convolutional-Based Attention Module (CBAM)

**Figure 2:**
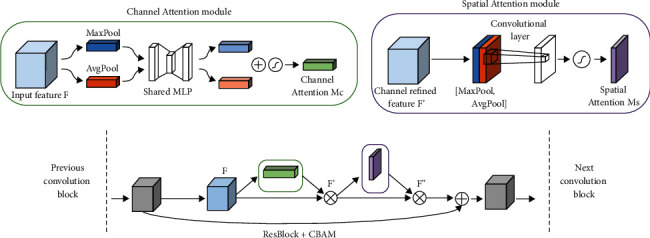
The sequential arrangement of CBAM channel and spatial attention submodules.

The strength of CNN in images and videos recognition is the availability of different convolutional kernels capable of extracting variations of features in the image. In this research, CBAM is adopted, which has two submodules: channel and spatial focused on detection tasks. Both attention submodules presented different functions. The channel attention submodule gives prominence to representative information provided by an input image. The spatial attention sub-module focuses on the representative region that contributes to the meaningfulness of the image. In addition, both submodules emphasised the concept of “What” and “Where.” Figure 2 shows the sequential order of these two submodules when processing information flow in the convolution block of the neural network.

The sequential order is chosen before parallel structure for both submodules, where input features are directed to channel attention followed by spatial attention. It was proven that sequential order generated better results [6].

In channel attention, average pooling and maximum pooling are applied separately. The input features will be directed into a multilayer perceptron (MLP) with only one hidden layer to generate channel attention maps. The element-wise summation will combine the two output maps to compute the channel attention sub-module. Equation (1) shows the representation of channel attention, Mc in symbols:

(1) $$\begin{array}{c} \text{Mc}\left(F\right)=\sigma \left(\text{MLP}\left(\text{AvgPool}\left(F\right)\right)+\text{MLP}\left(\text{MaxPool}\left(F\right)\right)\right), \end{array}$$

where *σ* refers to the sigmoid function applied, MLP is the multi-layer perceptron, AvgPool and MaxPool represent average pooling and maximum pooling, respectively.

Unlike channel attention, spatial attention submodules apply both average pooling and maximum pooling processes along the channel axis with a convolution layer to produce a spatial attention map. At this time, MLP is not implemented. The spatial attention Ms is shown in equation (2)rdf:

(2) $$\begin{array}{c} \text{Ms}\left(F^{\prime }\right)=\sigma \left(f\left(\left[\text{AvgPool}\left(F^{\prime }\right),\text{MaxPool}\left(F^{\prime }\right)\right]\right)\right), \end{array}$$

where *f* implies the convolution layer computation.

### 3.2. Integration of CBAM into Resnet-18 Architecture within Blocks and before Classifier

A residual block in ResNet-18 has a depth of two convolutional layers. “Within Blocks” refers to a method that plugged CBAM at every ResNet residual block in the neural network architecture [6]. The middle 16 convolutional layers in ResNet-18 will form 8 residual block structures. This structure inferred that the “Within Blocks” method integrated CBAM eight times between these consequent residual blocks. This CBAM, the residual network, can refine intermediate feature maps to vital information that better represents the input. While the “Before Classifier” technique integrated CBAM at the end part of the whole residual network, right before the average pool layer and fully connected (FC) layer. Through this implementation, CBAM will be used only once for every epoch of training, which has lower network complexity and consumes less computational cost compared to the “Within Blocks” method. After a given input in tensor, the format passes along all the convolutions in a residual block of CBAM-ResNet, transforming the final feature map into the average pool and FC layers. At this stage, only the last feature map will undergo refinement by CBAM. The refined outcome will then be classified to predict the label of input.

**Figure 3:**
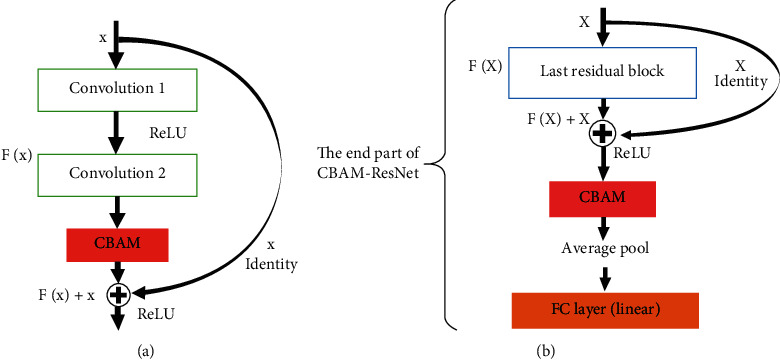
CBAM integrated into a residual block using: (a) “within blocks” and (b) “before classifier” method.

Figure 3(a) shows a single residual block in CBAM-ResNet, which visualises the exact place of the integrated attention module in residual network architecture using the “Within blocks” method. CBAM is implemented at the end of the residual function, *F* in its block.  Figure 3(b) shows the exact location of CBAM, which is the bottom part of CBAM-ResNet architecture using the “Before Classifier” method.

## 4. Experiment Settings and Results

This study implemented the modified CBAM-2DResNet for Malaysian static sign image recognition. The experiment was carried out to compare and evaluate the classification performance of CBAM integration methods into 2DResNet to complete static sign image recognition. A real-time static sign image recognition system using a webcam was built using the best CBAM-2DResNet trained model resulting from the comparison made.

### 4.1. Experiment Settings on Malaysian Static Signs Image Recognition

**Figure 4:**
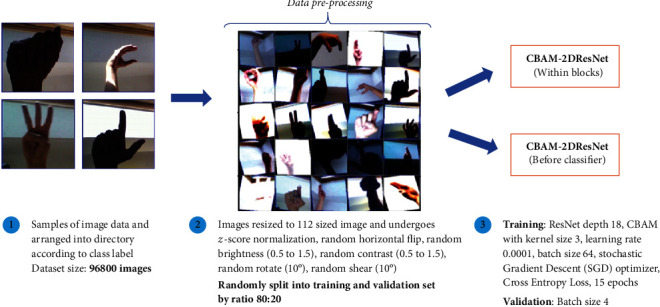
Summarised experimental procedures flow diagram on signs image recognition.

The development phase used Python programming language version 3.6 with Anaconda Spyder integrated development environment and utilised essential Python deep learning libraries such as Pytorch, Torchvision and CUDA Toolkit. This experiment was conducted in Google COLAB with Tesla K80 GPU for CBAM-2DResNet “Within Blocks” and “Before Classifier.” Figure 4 shows a summarised flow diagram of experimental procedures prepared for MSL static signs image recognition. Before starting the classification model training, data preprocessing and augmentation steps were set up on sign image data and several crucial neural network parameters. A collection of 96800 sign images was resized to 112 × 112 resolution images and normalised using z-score normalisation. Normalised images data were further processed with other images transformation operations, such as random image horizontal flip in 50% probability, random image brightness and contrast adjustment in the range between 0.5 and 1.5, random image rotation, and shear alteration within range ±10°. These data augmentation techniques applied can significantly improve the variation and diversity of the available data for training.

Random data splitting later separated these sign images into training and validation subset with ratio 8 : 2, which training subset take up 77440 images, and remaining 19360 images were in validation subset. Next, signs images in the training subset transformed to 4D tensors and loaded into both CBAM-2DResNet “Within Blocks” and “Before Classifier” to train for 15 epochs. Same network parameters were configured for training, such as learning rate = 0.0001, momentum = 0.9, CBAM kernel size with 3 × 3, and batch size of 64. The Stochastic Gradient Descent (SGD) optimiser was implemented, and Cross-Entropy Loss was adopted as a function to compute the training and validation losses over epochs. Validation was continued after training by choosing the best-trained classification model. A small validation batch size of 4 was utilised. The validation results required for the model's efficiency evaluation based on performance metrics were recorded and analysed.

### 4.2. Result of Signs Image Recognition Experiment Using CBAM -2DResNet

#### 4.2.1. Comparison of Training and Validation CBAM-2DResNet “Within Blocks”

**Figure 5:**
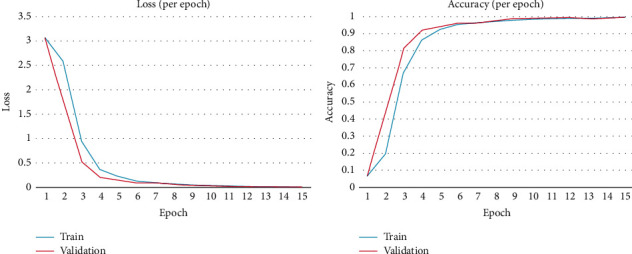
Training vs. validation loss and accuracy of CBAM-2DResNet “within blocks” and “before classifier.”

The comparison graph between the training and validation loss curve of CBAM-2DResNet “Within block” over 15 epochs is depicted in Figure 5. The training loss decreased steeply from 3.066 to 0.381 over epoch 1 to epoch 4 and responded to a very low decreasing rate from epoch 5 to epoch 15. While validation loss also decreased rapidly from 3.024 to 0.211 over epoch 1 to epoch 4 and diminished at a minimal rate afterwards. Both training and validation loss curves showed almost the same decreasing trends. The lowest training loss was recorded at epoch 15 with a value of 0.0252, while for lowest validation loss recorded was 0.0214 at the last epoch, in which both values were approximately the same. The minimal differences between training and validation losses indicated that this model achieved a good fit in learning.

In Figure 5, we also show the comparison graph between training and validation accuracy of CBAM-2DResNet “Within block” over 15 epochs. The training accuracy increased rapidly from 6.69% to 86.73% over epoch 1 to epoch 4 and responded to a slower increasing rate from epoch 5 to epoch 15, achieving the point of stability, while validation accuracy rapidly increased from 8.15% to 92.42% over epoch 1 to epoch 4 and turned to a moderate increasing rate on the epochs afterwards.

Both training and validation accuracy plots showed almost the same rising trends, in which the increments in the accuracy graph since epoch 10 were minor. The highest training accuracy was achieved at epoch 15 with 99.17%, while the highest validation accuracy attained was 99.29% at the last epoch scheduled. Both accuracies reached were comparable for this signs image dataset and almost similar. The minimal gap between training and validation accuracies implied that trained CBAM-2DResNet “Within block” was a well-fitted image recognition model.

#### 4.2.2. Comparison of Training and Validation CBAM-2DResNet “Before Classifier”

**Figure 6:**
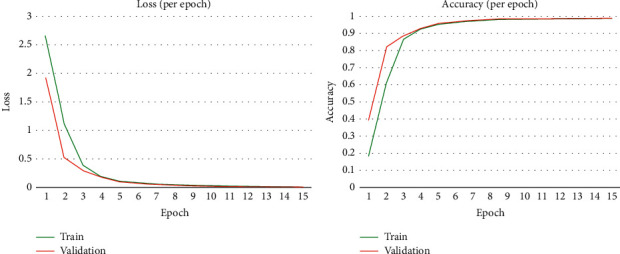
Training vs validation loss and accuracy of CBAM-2DResNet “before classifier.”

The training and validation loss comparison in the plotted graph for CBAM-2DResNet “Before classifier” over 15 epochs is shown in Figure 6. Both training and validation loss curves showed similar trends in decreasing, starting with a rapid decline followed by convergence to the point of stability. The training loss decreased steeply from 2.644 to 0.131 over epoch 1 to epoch 5 and responded to a minimal decrease starting from epoch 6 to epoch 15. Similarly, a rapid decrease from 1.913 to 0.188 over epoch 1 to epoch 4 was observed for validation loss which finished afterwards. Training loss recorded the lowest value of 0.0205 at epoch 14, while lowest validation loss was also recorded at the same epoch with value 0.0210, in which both loss values were much closed. This model possessed a good fit learning curve with the narrow differences between its final training and validation losses computed.

In Figure 6, we display the comparison plotted line curves between training and validation accuracy of CBAM-2DResNet “Before classifier” for 15 epochs. A rapid increase was observed for training accuracy from 18.80% to 95.62% over epoch 1 to epoch 5, followed by a slower increment rate on epoch afterwards. Accuracy for validation also increased quickly from 4.02% to 93.65% over epoch 1 to epoch 4 and slowed down later in the remaining epochs. A standard, increasing trend was noticed between both line graphs of training and validation. The accuracy value converged to the stabilisation point with a very low increment rate since epoch 6. The highest accuracy achieved for training and validation is 99.37% at epoch 15 and 99.39% at epoch 14, respectively. The comparable accuracies recorded by the training and validation phase implied that CBAM-2DResNet “Before classifier” after training was a good fit model with high predictive capacity on this dataset.

#### 4.2.3. CBAM-2DResNet Classification Report, Confusion Matrix, and *F*1-Score Bar Chart in “Within Blocks” and “Before Classifier”

Through Table 1, we list the precision, recall, and *F*1-score of CBAM-2DResNet “Within blocks” and “Before classifier” for each alphabet class on validation subset and the classes macro and weighted average generated with classification report function of Scikit-learn, the machine learning library of Python.

Where all values are within a range between 0.97 to 1. The proportion of instances that account for each class were taken into consideration when calculating the weighted average. Meanwhile, it was excluded in the calculation of the macro average. The recall, precision, and *F*1-score either in macro average or weighted average were reported with a value of 0.99 after round-off.

**Figure 7:**
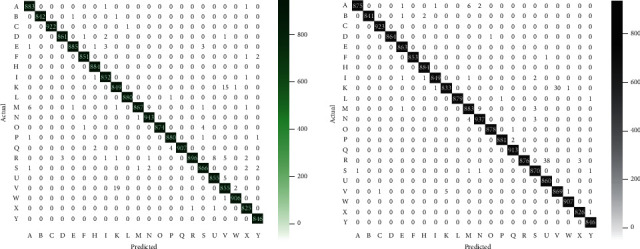
Confusion matrix plotted for CBAM-2DResNet “within blocks” and “before classifier.”

A multiclass confusion matrix was plotted for the classification result of CBAM-2DResNet “Within Blocks” and “Before Classifier” on the validation subset, as shown in Figure 7. This confusion matrix helped to give a closer look at the incorrect prediction that the classification model made. The false positives for each alphabet class were the green-coloured and grey-colour cells that were diagonally oriented in the confusion matrix. In contrast, other off-diagonal cells were the wrong predictions that were classified on other alphabet classes.

The model had its worst prediction on two classes, the alphabet “R” and “V,” with 21 misclassified instances for both classes. By taking alphabet “V” for further illustration, it had a correct prediction of 855 instances, where 19 instances were misclassified as “K” and 2 as “W,” out of its total of 876 sample images. Meanwhile, this model correctly classified another 2 classes, “H” and ‘Out of the 22 classes in the validation subset.

**Figure 8:**
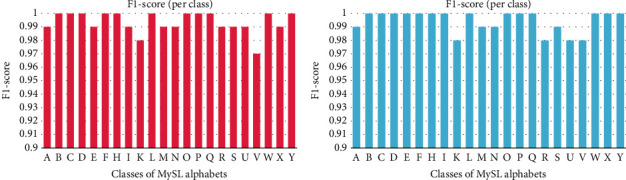
*F*1-score bar chart of CBAM-2DResNet “within blocks” and “before classifier.”

Figure 8 illustrates the *F*1-score bar chart for each alphabet class using CBAM-2DResNet “Within Blocks” and “Before Classifier” on validation subset. The *F*1-score of classes in “Within Blocks” were relatively high. 11 out of 22 classes reached the best value of 1.0, which included alphabets “B,” “C,” “D,” “F,” “H,” “L,” “O,” “P,” “Q,” “W” and “Y.” At the same time, alphabet “V” had the lowest *F*1-score, valued at 0.97. *F*1-score among classes in “Before Classifier” were all approximately to the best value 1. The 14 classes, alphabets “B,” “C,” “D,” “E,” “F,” “H,” “I,” “L,” “O,” “P,” “Q,” “W,” “X” and “Y” ranked the highest *F*1 score at 1. On the other hand, another 4 classes achieved 0.98, which were alphabet “K,” “R,” “U,” and “V.”

The “Before Classifier” misclassified an alphabet “R,” with a true positive of 876. Thirty-eight (38) instances were misclassified as “U,” 3 as “X,” out of its total 917 sample images. Among 22 alphabets in the validation subset, 7 classes contributed 100% correct predictions on all their instances, which are alphabets “C,” “E,” “H,” “Q,” “U,” “W,” and “Y.”

#### 4.2.4. Real-Time Malaysian Sign Language Recognition Using Image Recognition Technique

**Figure 9:**
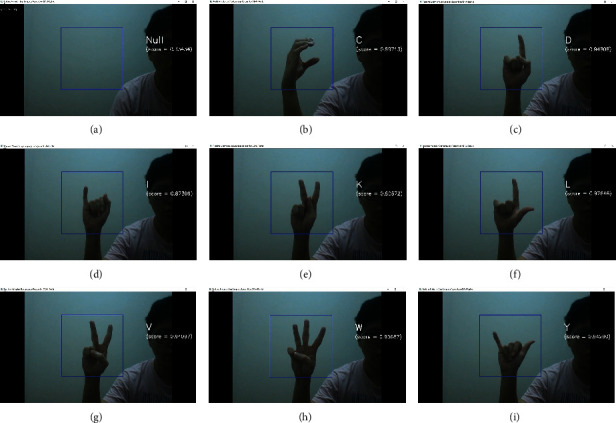
(a) “Null” classification result, (b) “C” correct classification with 0.897 confidence score, (c) “D” correct classification with 0.948 confidence score, (d) “I” correct classification with 0.873 confidence score, (e) “K” correct classification with 0.806 confidence score, (f) “L” correct classification with 0.976 confidence score, (g) “V” correct classification with 0.941 confidence score, (h) “W” correct classification with 0.957 confidence score, and (i) “Y” correct classification with 0.943 confidence score.

Figures 9(a)–9(i) presents the correct real-time classifications of certain MSL alphabet signs presented with their class predicted and confidence score, respectively. The best-trained CBAM-2DResNet “Before Classifier” was chosen as a classification model in building the real-time signs alphabet recognition application. This real-time application implemented with the OpenCV library provided a direct platform to evaluate the trained model through images extracted from webcam frames. Real-time signs images were extracted from the blue box region for every four frames captured through a webcam to feed as test inputs and returned the corresponding classification result to the user if the confidence score was higher than 0.5.

## 5. Discussion

CBAM-2DResNet implemented had capability in extracting important features such as hand or fingers from sign images.  Table 2 shows a comparison table comparing two different CBAM implementation methods by extracting the important results presented, including training duration, lowest validation loss, highest validation accuracy, *F*1-score achieved, and generalisation performance.

From the comparison table, CBAM-2DResNet “Within Bocks” and “Before Classifier” show good performance in signs image classification tasks for this MSL alphabet dataset. Similarly, both models achieved the *F*1-score of 0.99 computed with the classification report and reflected as good fit models in their generalisation performance. A minor difference of 0.0004 existed between the lowest validation loss values achieved by these two models. Their highest validation accuracy only varied for a 0.1% difference. However, these insignificant differences would not distinguish much on both models in terms of their classification efficiency.

**Figure 10:**
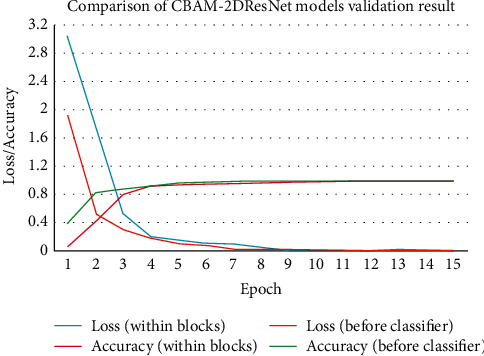
Comparison between CBAM-2DResNet “within blocks” and “before classifier” models in validation loss and accuracy.

The comparison graph of validation loss and accuracy between “Within Blocks” and “Before Classifier” models of the CBAM-2DResNet is given in Figure 10. It shows that the validation loss of the “Before Classifier” model is prone to decrease and converged faster than another model throughout all 15 epochs. Correspondingly, the validation accuracy of the “Before classifier” model also increased faster than model “Within Blocks.”

Noticed on classification performance for 22 alphabet classes, both CBAM-2DResNet “Within Blocks” and “Before Classifier” had the most significant number of wrong classified instances on class “R.” In real-time testing, it was observed that “Before Classifier” may sometimes do adjust classification with low confidence or misclassified certain alphabet signs with high similarities, such as hand signs “V” and “K” and hand signs “R” and “U.” The same misclassification issues can also be traced from the confusion matrix in Figure 7. Generally, CBAM-2DResNet “Before Classifier” is more efficient than CBAM-2DResNet “Within Blocks” in recognising static signs images.

## 6. Conclusion

This study is the pioneer to design and implement the CBAM-ResNet model into Malaysian Sign Language. Two experiments were conducted for static signs and dynamic signs using image recognition and video recognition techniques respectively. A Malaysian Sign Language video dataset consist of 19 dynamic signs was recorded. Two different CBAM integration attempts are applied in this research, which are known as “Within Blocks” and “Before Classifier” methods. The model achieved accuracy more than 90% with some variation. The CBAM-ResNet “Before Classifier” overall excels in recognition tasks on the images dataset. The CBAM-ResNet “Before classifier” is the best because it has a less computational cost and is 2.52 times faster in training than CBAM-ResNet “Within Blocks” in classification performance on video recognition experiments. This new approach in MSL recognition can be applied in real-time systems to help Malaysian signers in their daily communications.

During the dynamic sign34eds videos recognition and classification, an overfitting issue was observed. The overfitting may be because of the small data set; generally a dataset of over 100k samples is required to successfully optimise convolution kernels in CNNs architecture.

The concept of transfer learning can be applied in future research in coping with minor overfitting issues of CBAM-3DResNet in signs videos recognition. Another branch of artificial intelligence, Natural language processing (NLP) can extend this research to the next level, by constructing sentences with complete meaning from the recognised signs in video or through real-time. These interpretable sentences in either written or audio output could enhance the communication effectiveness between others and deaf-mutes. Finally, this model is also suitable to explore human action recognition.

## Figures and Tables

**Table 1:** Classification report of CBAM-2DResNet “within blocks.”

	Precision		Recall		*F*1-score		Support
	Within blocks	Before classifier	Within blocks	Before classifier	Within blocks	Before classifier	
A	0.99	0.99	1.00	1.00	0.99	0.99	885
B	1.00	1.00	1.00	1.00	1.00	1.00	844
C	1.00	1.00	1.00	1.00	1.00	1.00	932
D	1.00	1.00	1.00	1.00	1.00	1.00	865
E	1.00	1.00	0.99	0.99	0.99	0.99	893
F	1.00	1.00	1.00	1.00	1.00	1.00	854
H	1.00	1.00	1.00	1.00	1.00	1.00	884
I	0.99	0.99	1.00	1.00	0.99	0.99	854
K	0.97	0.97	0.98	0.98	0.98	0.98	865
L	1.00	1.00	1.00	1.00	1.00	1.00	881
M	1.00	1.00	0.98	0.98	0.99	0.99	886
N	0.99	0.99	1.00	1.00	0.99	0.99	944
O	1.00	1.00	0.99	0.99	1.00	1.00	879
P	0.99	0.99	1.00	1.00	1.00	1.00	883
Q	1.00	1.00	0.99	0.99	1.00	1.00	913
R	1.00	1.00	0.98	0.98	0.99	0.99	917
S	0.99	0.99	0.99	0.99	0.99	0.99	874
U	0.99	0.99	0.99	0.99	0.99	0.99	860
V	0.97	0.97	0.98	0.98	0.97	0.97	876
W	1.00	1.00	1.00	1.00	1.00	1.00	907
X	0.99	0.99	1.00	1.00	0.99	0.99	827
Y	0.99	0.99	100	100	1.00	1.00	846
Accuracy					0.99	0.99	19360
Macro avg.	0.99	0.99	0.99	0.99	0.99	0.99	19360
Weighted avg.	0.99	0.99	0.99	0.99	0.99	0.99	19360

**Table 2:** Comparison table for CBAM-2DResNet “within blocks” and “before classifier.”

Metrics	CBAM-2DResNet “within blocks”	CBAM-2DResNet “before classifier”
Training duration	16040.79 seconds	4860.23 seconds
Lowest validation loss	0.0214	0.0210
Highest validation accuracy	99.29%	99.39%
*F*1-score achieved	0.99	0.99
Generalisation performance	Good fit	Good fit

## Data Availability

The data that support the findings of this study are available upon request from the corresponding author.
